# *In vitro* and *in vivo* inhibition of human Fanconi anemia-associated head and neck squamous cell carcinoma by a novel nutrient mixture

**DOI:** 10.3892/ijo.2012.1639

**Published:** 2012-09-24

**Authors:** M.W. ROOMI, T. KALINOVSKY, N.W. ROOMI, A. NIEDZWIECKI, M. RATH

**Affiliations:** Dr Rath Research Institute, Santa Clara, CA 95050, USA

**Keywords:** Fanconi anemia, head and neck squamous cell carcinoma, nutrient mixture, tumor growth, matrix metalloproteinases, Matrigel invasion, cell migration

## Abstract

Head and neck squamous cell carcinoma (HNSCC) and acute myeloid leukemia are the major causes of mortality and morbidity in Fanconi anemia (FA) patients. The objective of this study was to investigate the antineoplastic activity of a novel antineoplastic nutrient mixture (NM) (containing lysine, proline, ascorbic acid and green tea extract) in human FA-associated HNSCC (FA HNSCC) *in vitro* and *in vivo*. The human FA HNSCC cell line, OHSU-974 (Fanconi Anemia Research Fund), was cultured in RPMI medium supplemented with 20% FBS and antibiotics. At near confluence, cells were treated in triplicate with various concentrations of NM: 0, 50, 100, 250, 500 and 1,000 *μ*g/ml. Cells were also treated with phorbol 12-myristate 13-acetate (PMA) to induce matrix metalloproteinase (MMP)-9 activity. Cell proliferation was detected by MTT assay, the secretion of MMPs by gelatinase zymo graphy, cell invasion through Matrigel, cell migration by a scratch test and morphology by hematoxylin and eosin (H&E) staining. *In vivo*, athymic male nude mice (n=12) were inoculated with 3×10^6^ OHSU-974 cells subcutaneously and randomly divided into 2 groups: group A was fed a regular diet and group B a regular diet supplemented with 1% NM. Four weeks later, the mice were sacrificed and their tumors were excised, weighed and processed for histological analysis. NM inhibited the growth of OHSU-974 tumors by 47% and tumor burden by 50%. At lower concentrations, NM demonstrated no effect on proliferation, but at 1,000 *μ*g/ml a 40% toxicity was observed. Zymography revealed the MMP-2 and PMA-induced MMP-9 secretion. NM suppressed the secretion of both MMPs in a dose-dependent manner, with a virtual inhibition at 500 *μ*g/ml. NM inhibited OHSU-974 cell invasion through Matrigel in a dose-dependent manner with a complete block at 1,000 *μ*g/ml. H&E staining showed no morphological changes below 500 *μ*g/ml. These results suggest that NM has potential therapeutic use in the treatment of human FA HNSCC.

## Introduction

Head and neck squamous cell carcinomas (HNSCCs), known for their aggressive growth and propensity to metastasize, are among the most common tumors that develop in patients with Fanconi anemia (FA) (1,2). In FA patients, although HNSCC is morphologically the same, its incidence and course are altered. By the age of 40 years, FA patients have a 14% chance of developing HNSCC in contrast to 0.038% in the general population (3). Furthermore, the associated risk factors of tobacco smoking and alcohol consumption that are associated with 85% of the non-FA-associated HNSCC (FA HNSCC) cases do not play as significant a role in FA; approximately 16% of FA HNSCC cases are associated with these risk factors (3). In patients with FA, HNSCC has been shown to be more aggressive with early lymph node metastases and early soft tissue invasion resulting in poorer prognoses than in HNSCC patients without FA (3). Secondary primary tumors occur in 63% of FA patients compared to only 15% in non-FA patients (3). Furthermore, the 2-year overall survival is only 49% in FA patients compared to 70% in non-FA patients (3). The most frequent location of HNSCC is in the oral cavity (65%) compared to the larynx, hypoharynx and oropharynx, each at 10%, which differs from HNSCC in the general population. Due to significant toxic sequelae from the use of radiation therapy and/or chemotherapy in FA patients, surgical treatment is the main modality used. HNSCC in the general population is treated with radiation, chemotherapy and surgery. The highly metastatic potential of HNSCC in FA patients and inadequate treatment methods, leading to poor outcomes, create an urgent need to develop more effective and less toxic treatment alternatives.

We previously developed strategies to inhibit cancer development and its spread using naturally occurring nutrients, such as lysine, proline, ascorbic acid and green tea extract. This nutrient mixture (NM) has exhibited synergistic anticancer activity *in vivo* and *in vitro* in a number of cancer cell lines by inhibiting cancer cell growth, matrix metalloproteinase (MMP) secretion, invasion, metastasis and angiogenesis (4–6). In the present study, we examine the effect of this NM on the human OHSU-974 FA HNSCC cell line *in vivo*, in athymic nude mice bearing HNSCC xenografts, as well as *in vitro*, evaluating cell viability, MMP secretion, invasion and migration.

## Materials and methods

### 

#### Cancer cell line and culture

The human OHSU-974 FA HNSCC cell line was obtained from the Fanconi Anemia Research Fund, Oregon Health and Science University, Portland, OR, USA. FA HNSCC cells were maintained in RPMI medium supplemented with 20% fetal bovine serum (FBS), 100 U/ml penicillin and 100 *μ*g/ml streptomycin. The media and sera used were obtained from the American Type Culture Collection (ATCC), and the antibiotics (penicillin and streptomycin) were from Gibco-BRL (Long Island, NY, USA).

#### Composition of the NM

The NM was composed of the following at the indicated ratios: vitamin C (as ascorbic acid and as Mg, Ca, and palmitate ascorbate) 700 mg; L-lysine 1,000 mg; L-proline 750 mg; L-arginine 500 mg; N-acetylcysteine 200 mg; standardized green tea extract [derived from green tea leaves, was obtained from US Pharma Lab Inc. (Santa Clarita, CA, USA); the certificate of analysis indicated the following characteristics: total polyphenol 80%, catechins 60%, epigallocatechin gallate (EGCG) 35% and caffeine 1.0%] 1,000 mg; selenium 30 *μ*g; copper 2 mg; and manganese 1 mg.

### In vivo studies

#### Animals

Male athymic mice (NCr-nu/nu), approximately 5 weeks of age on arrival, were purchased from Simonsen Laboratories (Gilroy, CA, USA) and maintained in microisolator cages under pathogen-free conditions on a 12-h light/12-h dark schedule for a week. All procedures were performed according to humane and customary care and use of experimental animals and followed a protocol approved by the internal institutional animal safety review committee of our institution.

#### Experimental design

After housing for a week, the mice (n=12) were inoculated subcutaneously with 3×10^6^ OHSU-974 cells in 0.2 ml phosphate-buffered saline (PBS) and 0.1 ml Matrigel (BD Bioscience, Bedford, MA, USA). After injection, the mice were randomly divided into 2 groups; group A mice were fed regular Purina mouse chow and group B the regular diet supplemented with 1% NM (w/w). The regular diet was Laboratory Rodent Diet 5001 from Purina Mills (Gray Summit, MO, USA) LLC/Test Diet. The 1% NM diet was milled and pressed by Purina Mills, LLC and generated by Vitatech (Tustin, CA, USA). During the study, the mice consumed, on average, 4 g of their respective diets per day. Thus, the supplemented mice received approximately 40 mg of NM per day. After 4 weeks, the mice were sacrificed and their tumors were excised and processed for histological analysis. Dimensions (length and width) of tumors were measured using a digital caliper, and the tumor burden was calculated using the following formula: 0.5 × length × width. The mean weight of the mice at the initiation and termination of the study did not differ significantly between the groups.

#### Histological analysis

Tissue samples were fixed in 10% buffered formalin. All tissues were embedded in paraffin and cut at 4–5 microns thick. Sections were deparaffinized through xylene and graduated alcohol series to water and stained with hematoxylin and eosin (H&E) for evaluation using a standard light microscope.

### In vitro studies

#### Cell culture

The human OHSU-974 HNSCC cells were grown in RPMI, supplemented with 20% FBS, penicillin (100 U/ml) and streptomycin (100 mg/ml) in 24-well tissue culture plates (Costar, Cambridge, MA, USA). Cells were incubated in 1 ml of medium at 37°C in a tissue culture incubator equilibrated with 95% air and 5% CO_2_. At near confluence, the cells were treated with the NM, dissolved in medium and examined at 0, 50, 100, 250, 500, and 1,000 *μ*g/ml in triplicate at each dose. Phorbol 12-myristate 13-acetate (PMA), 100 ng/ml was added to cells to induce MMP-9 secretion. The plates were then returned to the incubator.

#### MTT assay

Cell viability was evaluated by [3-(4,5-dimethylthiazol-2-yl) 2,5-diphenyl tetrazolium bromide] (MTT) assay, a colorimetric assay based on the ability of viable cells to reduce a soluble yellow tetrazolium salt MTT to a blue formazan crystal by mitochondrial succinate dehydrogenase activity of viable cells. This test is a good index of mitochondrial activity and thus of cell viability. After 24 h of incubation, the cells were washed with PBS and 500 *μ*l of MTT (Sigma #M-2128) 0.5 mg/ml in medium was added to each well. After the addition of MTT (0.5 mg/ml) the plates were covered and returned to the 37°C incubator for 2 h, the optimal time for formazan product formation. Following incubation, the supernatant was carefully removed from the wells, the formazan product was dissolved in 1 ml dimethylsulphoxide (DMSO), and absorbance was measured at 570 nm in a BioSpec 1601, Shimadzu spectrometer. The OD_570_ of the DMSO solution in each well was considered to be proportional to the number of cells. The OD_570_ of the control (treatment without supplement) was considered 100%.

#### Gelatinase zymography

Gelatinase zymography was performed in 10% Novex Pre-Cast SDS polyacrylamide gel (Invitrogen) in the presence of 0.1% gelatin under non-reducing conditions. The culture media (20 *μ*l) were mixed with sample buffer and loaded for SDS-PAGE with Trisglycine-SDS buffer, as suggested by the manufacturer (Novex). Samples were not boiled prior to electrophoresis. Following electrophoresis, the gels were washed twice in 2.5% Triton X-100 for 30 min at room temperature to remove SDS. The gels were then incubated at 37°C overnight in substrate buffer containing 50 mM Tris-HCl and 10 mM CaCl_2_ at pH 8.0 and stained with 0.5% Coomassie Blue R250 in 50% methanol and 10% glacial acetic acid for 30 min and destained. Upon renaturation of the enzyme, the gelatinases digested the gelatin in the gel, producing clear bands against an intensely stained background. Protein standards were run concurrently and approximate molecular weights were determined by plotting the relative mobilities of known proteins.

#### Matrigel invasion

Invasion experiments were conducted using Matrigel (Becton-Dickinson) inserts in 24-well plates. Suspended in medium, OHSU-974 cells were supplemented with nutrients, as specified in the design of the experiment and seeded on the insert in the well. Thus both the medium on the insert and in the well contained the same supplements. The plates with the inserts were then incubated in a culture incubator equilibrated with 95% air and 5% CO_2_ for 24 h. After incubation, the medium was withdrawn from the wells. The cells on the upper surface of the inserts were gently scrubbed away with cotton swabs. The cells that had penetrated the Matrigel membrane and migrated onto the lower surface of the Matrigel were stained with H&E and visually counted under a microscope.

### Cell migration: scratch test

To examine cell migration, a 2-mm wide single uninterrupted scratch was made from the top to bottom of culture plates of OHSU-947 cells grown to confluence. Culture plates were washed with PBS and incubated with NM in medium and examined at 0, 50, 100, 250 and 500 *μ*g/ml, in triplicate at each dose for 24 h. Cells were washed with PBS, fixed and stained with H&E and photomicrographs were obtained.

#### Morphology: H&E

The morphology of the cells cultured for 24 h in the test concentrations of NM was evaluated by H&E staining and observed and photographed under a microscope.

#### Statistical analysis

The results are expressed as the means ± SD. Data were analyzed by an independent sample t-test. Pearson’s correlation co-efficients were determined for toxicity and invasion correlations to the NM concentration using MedCalc software (Mariakerke, Belgium).

## Results

### In vivo studies

#### Tumor growth and burden

**Figure 1:**
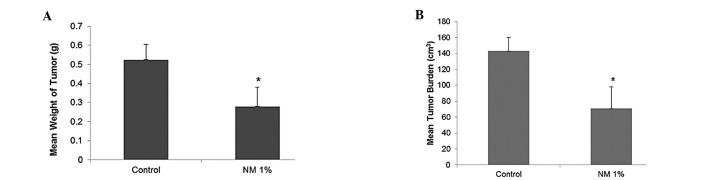
(A) Effect of 1% NM dietary supplementation on mean tumor weight of OHSU-974 xenografts in male nude mice injected with 3×10^6^ OHSU-974 cells (^*^p=0.005 with respect to control). (B) Effect of 1% NM dietary supplementation on tumor burden of OHSU-974 xenografts in male nude mice (^*^p=0.005 with respect to control).

NM strongly inhibited the growth of OHSU-974 xenografts in nude mice. Mean tumor weight was inhibited by 47% (p=0.0009) with NM 1% dietary supplementation, as shown in Fig. 1A and tumor burden was inhibited by 50% (p=0.0003), as shown in Fig. 1B.

#### Histological analysis

**Figure 2:**
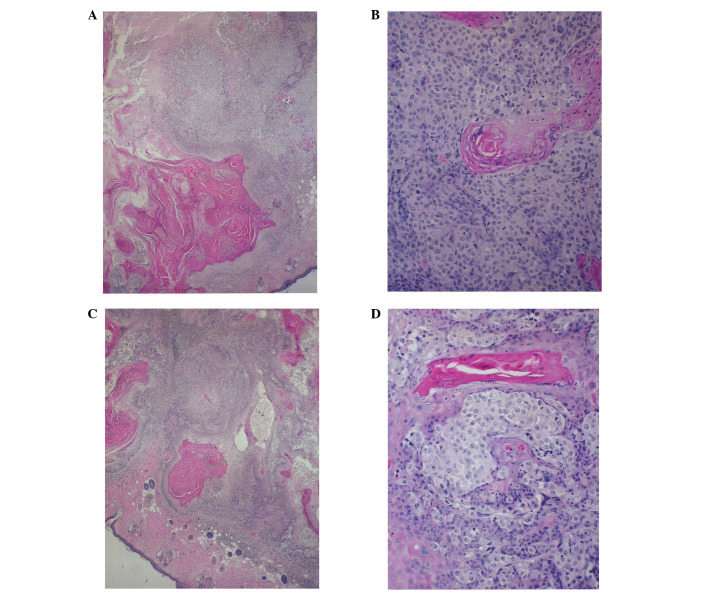
Histopathology of tumors. Representative tumors from (A) control, ×40; (B) control, ×200 and 1% NM-supplemented mice; (C) 1% NM, ×40; (D) 1% NM, ×200.

Histologically, the tumors from both groups were composed of solid nests of large, irregularly round, ulcerated, skin subcutaneous masses, consistent with squamous cell carcinoma. Tumors from the control and NM-supplemented mice were similar morphologically, although the tumors from the NM-supplemented mice were significantly smaller in size (Fig. 2).

### In vitro studies

#### Cytotoxicity

**Figure 3:**
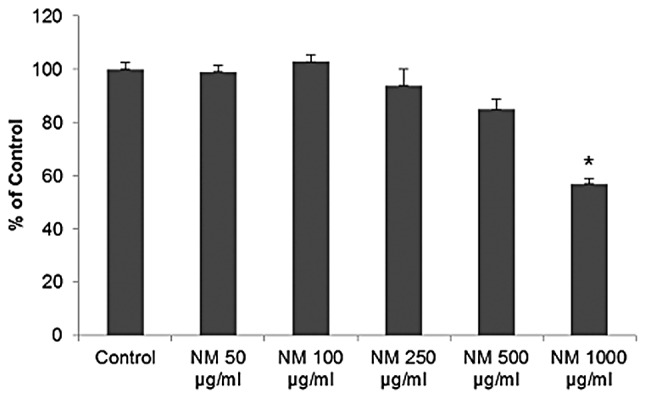
Effect of NM on viability of OHSU-974 cells, MTT 24 h (^*^significance of at least p=0.005 with respect to control).

NM exhibited no significant toxicity to OHSU-974 HNSCC cells *in vitro* at lower concentrations. However, a cytotoxicity of 15 (p=0.005) and 40% (p<0.001) at 500 and 1,000 *μ*g/ml was observed, respectively, compared to the control, as shown in Fig. 3.

#### Gelatinase zymography

**Figure 4:**
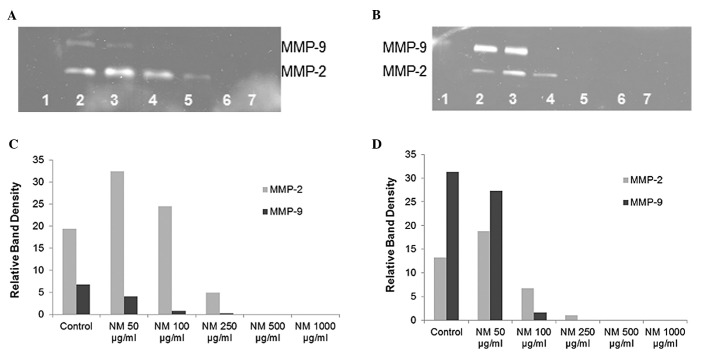
Effect of NM on MMP-2 and MMP-9 secretion in (A) normal OHSU-974 cells (B) PMA (100 ng/ml)-treated OHSU-974 cells. Lane 1, markers; lane 2, control; lanes 3–7, NM 50, 100, 250, 500 and 1,000 *μ*g/ml, respectively. Densitometry analysis of (C) uninduced and (D) PMA-treated OHSU-974 cells.

Gelatinase zymography demonstrated MMP-2 and MMP-9 secretion by normal and PMA-treated OHSU-947 cells. NM inhibited the secretion of both MMPs in a dose-dependent manner with virtual total inhibition of MMP-9 and MMP-2 at 500 *μ*g/ml, as shown in Fig. 4. MMP-2 secretion by normal OHSU-947 cells was inhibited by 75% at 250 *μ*g/ml NM and by 100% at 500 *μ*g/ml and 1,000 *μ*g/ml NM (linear trend, R^2^=0.6863) and the secretion of MMP-2 by PMA-treated cells was inhibited by 50% at 100 *μ*g/ml NM, 99% by 250 *μ*g/ml NM and by 100% at 500 *μ*g/ml and 1,000 *μ*g/ml NM (linear trend, R^2^=0.7578). MMP-9 secretion by normal OHSU-947 cells was inhibited by 88% at 100 *μ*g/ml NM, 96% by 250 *μ*g/ml NM and by 100% at 500 *μ*g/ml and 1,000 *μ*g/ml NM (linear trend, R^2^=0.7898) and the secretion of PMA-treated cells was inhibited by 95% at 100 *μ*g/ml NM and by 100% at 250, 500 and 1,000 *μ*g/ml NM (linear trend, R^2^=0.7324).

#### Matrigel invasion

**Figure 5:**
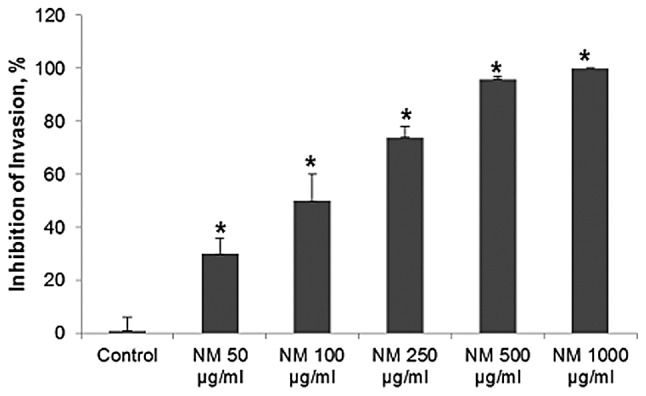
Effect of NM on Matrigel invasion of OHSU-974 cells (^*^significance of at least p=0.001 with respect to control).

**Figure 6:**
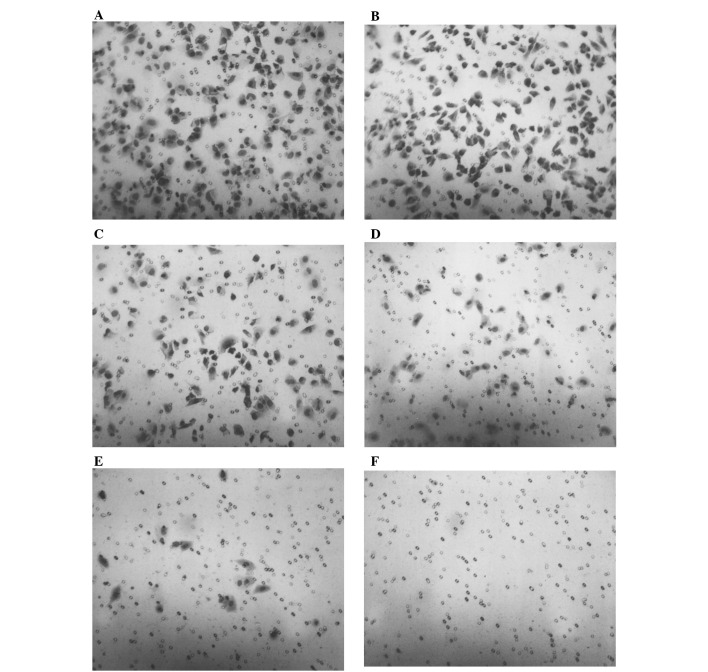
Effect of NM on Matrigel invasion: photomicrographs of (A) control, (B) 50 *μ*g/ml NM, (C) 100 *μ*g/ml NM, (D) 250 *μ*g/ml NM, (E) 500 *μ*g/ml NM, and (F) 1,000 *μ*g/ml NM.

NM significantly inhibited OHSU-974 cell invasion through Matrigel in a dose-dependent manner, with 30% (p=0.003) inhibition at 50 *μ*g/ml, 50% (p=0.002) at 100 *μ*g/ml, 74% (p<0.0001) at 250 *μ*g/ml, 96% (p<0.0001) at 500 *μ*g/ml and 100% (p<0.0001) at 1,000 *μ*g/ml, as shown in Figs. 5 and 6. There was a significant negative correlation between the NM concentration and the number of OHSU-974 cells that had invaded/migrated through Matrigel (r=−0.9715, p<0.0001).

#### Cell migration: scratch test

**Figure 7:**
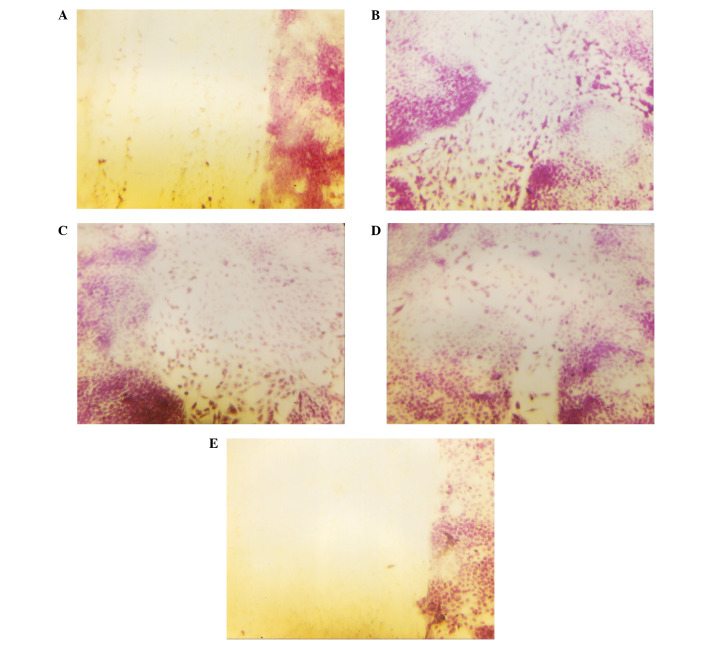
Effect of NM on migration: scratch test showing (A) control, 0 h; (B) control, 24 h; (C) 50 *μ*g/ml NM, 24 h; (D) 100 *μ*g/ml NM, 24 h; and (E) 250 *μ*g/ml NM, 24 h.

NM reduced cell migration in a dose-dependent manner, with a complete block of OHSU-974 cells at 250 *μ*g/ml. Photomicrographs of the results from the scratch tests of OHSU-974 cells are shown in Fig. 7.

#### Morphology: H&E staining

**Figure 8:**
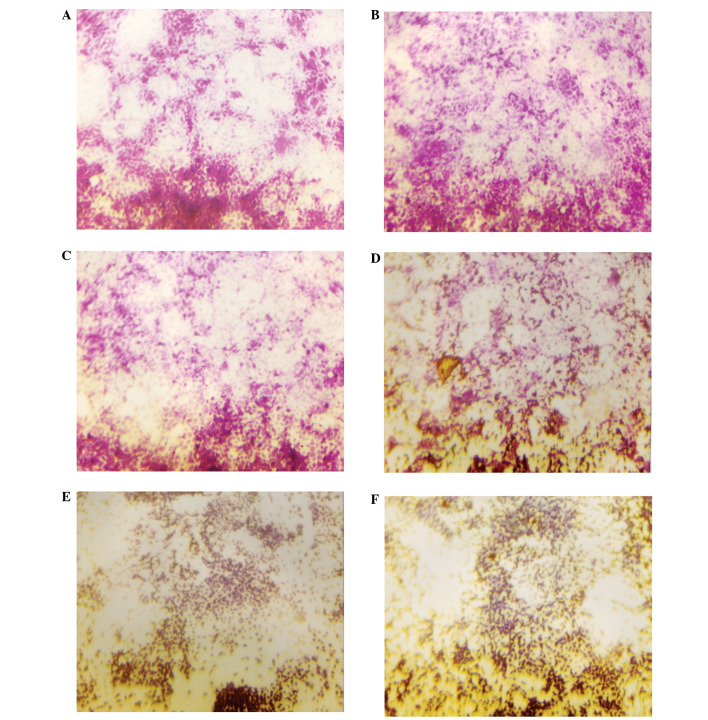
Effect of NM on morphology of OHSU-974 cells: H&E staining of (A) control, (B) 50 *μ*g/ml NM, (C) 100 *μ*g/ml NM, (D) 250 *μ*g/ml NM, (E) 500 *μ*g/ml NM, and (F) 1,000 *μ*g/ml NM.

No morphological changes were observed following H&E staining below 500 *μ*g/ml, as shown in Fig. 8.

## Discussion

The results of the *in vivo* study of human HNSCC xenografts in immune impaired (athymic) nude mice demonstrated a significant suppression of HNSCC OHSU-974 tumor growth (47% inhibition of mean tumor weight and 50% inhibition of mean tumor burden with 1% NM dietary supplementation). The results from the cellular proliferation study support the *in vivo* findings, as NM showed an increased toxicity in OHSU-974 cells in a dose-dependent manner, with 40% inhibition of cell growth exposed to 1,000 *μ*g/ml NM.

Growing tumors become hypoxic and acidotic beyond the size of 2 mm and secrete several growth factors to stimulate local angiogenesis. In a previous study, we demonstrated that NM significantly (p<0.05) reduced bFGF-induced angiogenesis [utilizing a chorioallantoic membrane (CAM) assay in chick embryos], and decreased the human U2OS osteosarcoma cell expression of VEGF, angiopoietin-2, bFGF, PDGF and TGFβ-1 (4).

The invasion of host tissues is dependent on tumor cell adhesion, cell migration and the proteolytic degradation of the extracellular matrix (ECM) by MMPs (7). MMPs, particularly MMP-2 and MMP-9, are prognostic markers for survival and metastatic potential in head and neck squamous carcinomas. In an examination of genolytic activity in human oral squamous cell carcinoma tissues, Kawamata *et al*(8) observed increased activity of pro-MMP-9 and active MMP-2 in cancer cell nests compared with normal surrounding gingival tissue and significantly higher MMP-2 activity in metastatic cancer cell nests. Patel *et al*(9) reported a significant elevation of latent, active and total forms of MMP-2 and MMP-9 in malignant tissue compared with adjacent normal tissues in oral cancer patients. In addition, MMP-2 correlated with lymph node metastatic development (9). In an examination of a group of patients with early stage oral squamous cell carcinoma, Katayama *et al*(10) found that patients who developed regional lymph node and/or distant metastasis showed significantly increased MMP-9 and TIMP metallopeptidase inhibitor-2 (TIMP-2) expression compared to patients without any tumor metastasis. In addition, the expression of MMP-9 and TIMP-2 correlated with worst cause-specific survival. Reidel *et al*(11) found that MMP-9 expression in patients with HNSCC correlated with poor survival, high VEGF expression and higher mean vessel density compared to MMP-9-negative tumors, suggesting that MMP-9 functions as a regulator of tumor angiogenesis supporting endothelial cell invasion in human head and neck cancer. Kurahara *et al*(12) demonstrated a significant decrease in ECM staining (indicating loss of ECM) in invasive and metastatitc cases of oral squamous cell carcinoma with increased expression of MMP-1, MMP-2 and MMP-9.

The results from our *in vitro* study of OSH-947 HNSCC cells demonstrated a potent, significant suppression of invasive parameters by the NM. NM inhibited MMP-2 and MMP-9 secretion with a complete block of both at 500 *μ*g/ml and 100% inhibition of invasion of cells through Matrigel at 1,000 *μ*g/ml. The migration of cells using a scratch test showed total block at 250 *μ*g/ml NM. In a previous study of HNSCC FaDu cells, NM was found to inhibit xenograft tumor growth and invasive parameters (13).

NM was formulated by defining critical physiological targets in cancer progression and metastasis, such as ECM integrity and MMP activity. Adequate supplies of ascorbic acid and the amino acids, lysine and proline, ensure proper synthesis and hydroxylation of collagen fibers for optimal ECM structure. Manganese and copper are also essential for collagen formation. Lysine, a natural inhibitor of plasmin-induced proteolysis, plays an important role in ECM stability (14,15). Green tea extract has been shown to modulate cancer cell growth, metastasis, angiogenesis and other aspects of cancer progression (16–20). N-acetylcysteine has been shown to modulate MMP-9 and the invasive activities of tumor cells (21,22). Selenium has been shown to inhibit MMP secretion, tumor invasion, and the migration of endothelial cells through the ECM (23). Ascorbic acid demonstrates cytotoxic and antimetastatic effects on malignant cell lines (25–28) and cancer patients have been found to have low levels of ascorbic acid (29,30). Low levels of arginine, a precursor of nitric oxide (NO), can limit the production of NO, which has been shown to predominantly act as an inducer of apoptosis (31).

Current treatment methods available for FA-associated cancers are generally ineffective and particularly toxic to these patients. Thus, there is a need for development of effective therapeutic agents for these cancers with minimal toxicity. In this study, our results demonstrated that NM significantly inhibited the growth and tumor burden of the OHSU-974 FA HNSCC cell line *in vivo*. In addition, invasive parameters, such as OHSU-974 cell line MMP-2 and -9 secretion and invasion were significantly inhibited by NM *in vitro*. These findings suggest the potential use of NM in the treatment of FA HNSCC. Furthermore, in contrast to the toxic side-effects of current chemotherapy, the NM has been shown to be a safe therapeutic agent. In a previous *in vivo* study addressing safety issues, we found that gavaging adult female ODS rats (weighing 250–300 g) with the NM (at 30, 90 or 150 mg per day for 7 days), had no adverse effects on vital organs (heart, liver and kidney), and on associated functional serum enzymes, indicating that this mixture is safe to use even at these high doses, which far exceed the normal equivalent dosage of the nutrient (32).
